# Impact of Pulsed‐Field Ablation on the Left Atrial Appendage Diameter—Insights From Intra‐Procedural Echocardiography

**DOI:** 10.1111/jce.70111

**Published:** 2025-09-19

**Authors:** Nándor Szegedi, Piotr Gardziejczyk, Zoltán Salló, Gábor Orbán, Márton Boga, Paweł Szymkiewicz, Marta Skowrońska, Patrik Tóth, Ferenc Komlósi, Béla Merkely, Ewa Wlazłowska‐Struzik, László Gellér, Jakub Baran, Dhiraj Gupta

**Affiliations:** ^1^ Heart and Vascular Center Semmelweis University Budapest Hungary; ^2^ Division of Clinical Electrophysiology, Department of Internal Medicine and Cardiology Medical University of Warsaw Warsaw Poland; ^3^ Liverpool Heart and Chest Hospital Liverpool UK

**Keywords:** diameter, edema formation, intracardiac echocardiography, left atrial appendage, pulsed‐field ablation, transesophageal echocardiography

## Abstract

**Introduction:**

Concomitant pulmonary vein isolation (PVI) and left atrial appendage (LAA) occlusion (LAAO) have become frequently used therapies. Pulsed‐field ablation (PFA), will likely be used for combined PVI plus LAAO procedures. However, there may be concerns regarding the malposition of the LAAO device attributed to the potential tissue edema after PVI.

**Aim:**

We aimed to compare the LAA's size before and after PVI performed with the pentaspline catheter, measured intraprocedural by intracardiac echocardiography or transesophageal echocardiography.

**Methods:**

We conducted a multicenter, prospective, observational study investigating PVI using the Farapulse system. The anteroposterior diameter of the left‐sided pulmonary vein (LPV), left atrial ridge, and LAA were measured before and after the PFA.

**Results:**

We enrolled 91 patients aged 63 ± 10 years, 36% were women, and 47% had paroxysmal AF. The most common comorbidities was hypertension (59%). Procedure time and left atrial dwell time were 65 (52–80) min, and 27 (24–32) min, respectively. The diameter of the LAA was not different before and after the PVI (15 [13–18] and 16 [13–19], respectively; *p* = 0.756). On the other hand, the diameter of the left atrial ridge (7 [6–8] and 8 [6–8]) and the LPV (13 [10–15] and 13 [11–15]) was smaller before ablation compared to the diameter after PVI (*p* < 0.0001 for both). No major complications occurred.

**Conclusion:**

LAA anteroposterior diameter does not change after PVI with PFA. Although there is a significant change in the anteroposterior diameters of the LPV and the left atrial ridge, it does not seem clinically relevant. If significant edema formation is detected in a single case after PVI, postponing the LAAO procedure should be considered.

## Graphical abstract

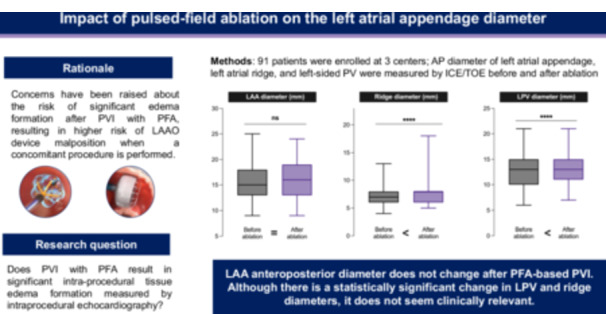

## 1 Introduction

Atrial fibrillation (AF) is the most common cardiac arrhythmia in adults and is associated with a higher risk of stroke, heart failure and death. Rhythm control therapy has recently been shown to be superior compared to rate control [1]. The most effective rhythm control therapy is catheter ablation (e.g., pulmonary vein isolation, PVI) [2], which may even result in better outcomes in terms of mortality in selected patient populations [3, 4].

One of the most serious complications of AF is stroke. Both ischemic and hemorrhagic stroke may occur, either as a consequence of the AF itself or its treatment [5, 6]. Oral anticoagulation is recommended in patients with elevated embolic risk, even after successful PVI. Percutaneous left atrial appendage (LAA) occlusion (LAAO) is a device‐based treatment that may be considered in patients with AF and contraindications for long‐term anticoagulation to prevent ischemic stroke and thromboembolism [5].

In some cases, both PVI and LAAO are indicated. Performing two separate procedures may carry an additional risk of complications (e.g., vascular adverse events and cardiac tamponade) [7, 8]. Thus, in these patients, concomitant PVI and LAAO present an attractive treatment option, the safety and efficacy of which has recently been confirmed by the OPTION trial. In the OPTION study, PVI was performed mainly with radiofrequency or cryo energy [9]. As PFA is becoming the leading source of energy for PVI [10, 11, 12], it will likely be used more frequently for concomitant PVI and LAAO procedures in the near future.

Smaller peri‐device leaks may be present in a non‐negligible percentage of patients undergoing LAAO, resulting in a higher thromboembolic and bleeding risk [13]. The acute edema formation after PFA may alter the LAA orifice size, leading to a potential risk of choosing the wrong LAAO device size [14]. Whether PF (pulsed‐field) applications change the LAA size has not yet been investigated.

We aimed to compare the size of the LAA and its neighboring structures before and after PVI performed with the pentaspline catheter, measured intraprocedural by intracardiac echocardiography (ICE) or transesophageal echocardiography (TOE).

## 2 Methods

### 2.1 Patient Population

In the current multicenter, prospective, observational study, we enrolled consecutive patients undergoing PVI procedures using the Farapulse PFA system (Boston Scientific, Marlborough, MA). Inclusion and exclusion criteria are listed in the Supporting Information. System components are the Farastar generator, which delivers a 2 kV biphasic, bipolar pulsed electric field, the Faradrive steerable sheath, and the Farawave pentaspline ablation catheter. Indications for AF ablation were in accordance with the current guidelines. Patients provided written, informed consent to the ablation procedure, data retrieval, and analysis. Ethics approval was obtained for all three centers' local ethics committees.

### 2.2 Ablation Procedure

Based on the centers' local practice, the catheter ablation was performed under general anesthesia or conscious sedation [15]. Femoral venous access and a single transseptal puncture were used for all procedures. Intravenous heparin was administered according to body weight and guided by ACT measurements; the target value was a minimum of 300 s. PVI was performed as described previously by applying four PF applications in basket and four in flower configurations for each PV [16]. Left‐sided PVs were ablated first, followed by the right‐sided PVs. If catheter‐tissue contact was deemed insufficient, more than eight applications per PV were permitted. At the end of the procedure, hemostasis of the puncture site was ensured by a figure of eight suture.

### 2.3 Intraprocedural Echocardiography Measurements

**Figure 1:**
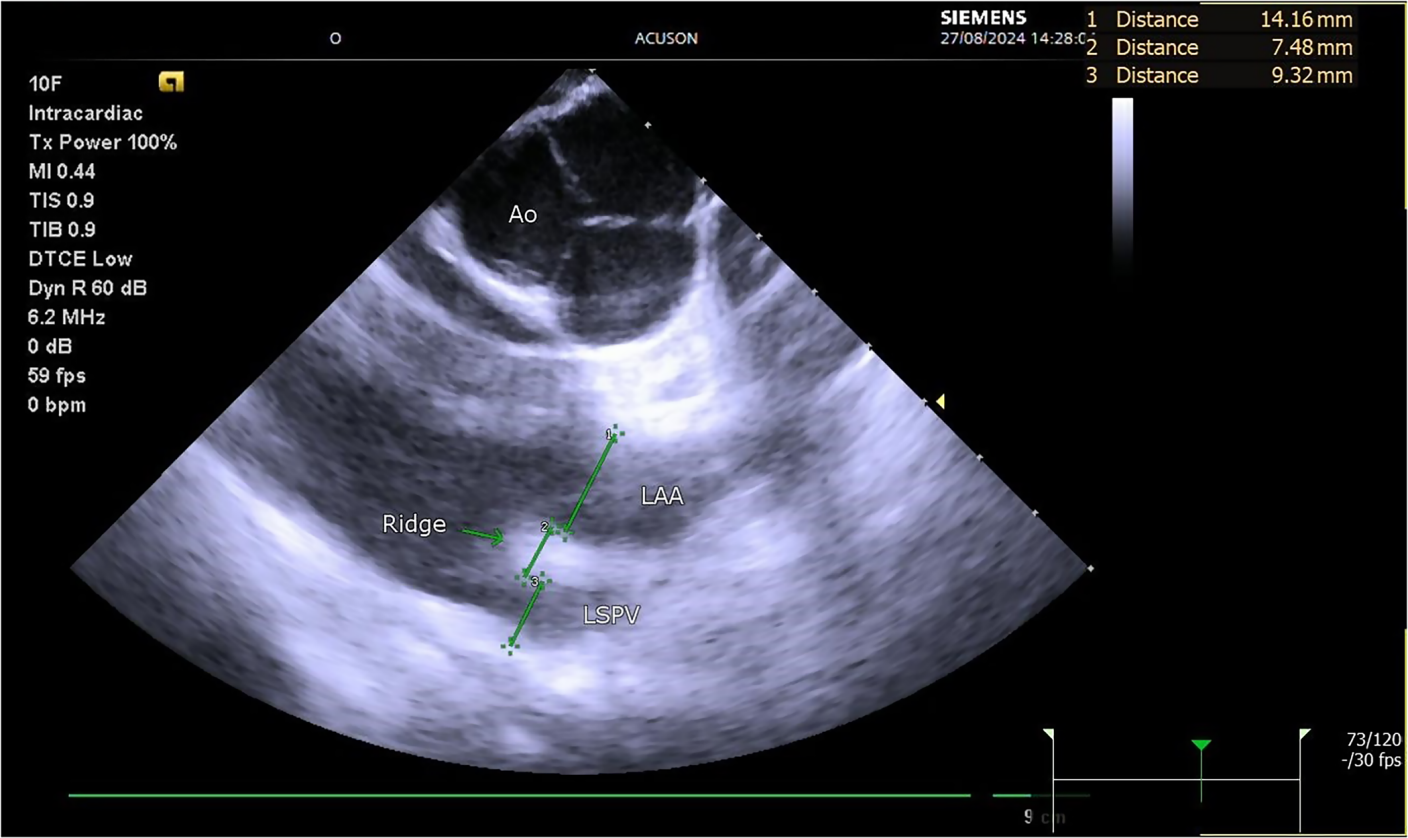
Intracardiac echocardiography measurements from the RVOT. Abbreviations: Ao, aorta; LAA, left atrial appendage; LSPV, left superior pulmonary vein.

For procedures where ICE was used, a 10‐F ICE catheter (AccuNav, Biosense Webster Inc, Diamond Bar, CA, USA) was introduced through the femoral vein into the proximal part of the right ventricular outflow tract to visualize the left atrium at the level of the left superior pulmonary vein (LSPV) or long left common trunk, the LA ridge and LAA orifice. The anteroposterior diameter of all three structures was measured (see Figure 1) before the PFA and at the end of the procedure. We performed the measurements when the aortic valve closed to ensure all parameters were obtained in the same cardiac cycle. For patients where TOE was used, the same measurements were made in the mid‐esophageal 90° left view. To assess interobserver variability, ICE measurements of 15 patients were repeated by another researcher.

### 2.4 Statistical Analysis

Continuous variables are presented as mean and standard deviation or median and interquartile ranges where appropriate, and their distribution was tested with the Shapiro–Wilk test. Wilcoxon signed‐rank test was used for paired group comparison. Categorical variables are presented as frequency and percentage. Interobserver reliability was assessed using a two‐way random‐effects intraclass correlation coefficient (ICC). Statistical analyses were performed using GraphPad Prism 8 (GraphPad Softwares Inc., USA). A two‐tailed *p*‐value of < 0.05 was considered statistically significant.

## 3 Results

We enrolled 91 patients in the study; age 63 ± 10 years, 33 (36%) were women, and 43 (47%) had paroxysmal AF. The most common comorbidities were hypertension (59%) and diabetes (32%). A detailed description of the study population is shown in Table 1.

**Table 1:** Baseline characteristics of the study population.

Parameter	Population (*n* = 91)
Age, years; mean (SD)	63 (10)
Female sex, *n* (%)	33 (36)
BMI, kg/m2; mean (SD)	30.5 (4.8)
Paroxysmal AF, *n* (%)	43 (47)
EHRA symptom score; median (IQR)	2 (2–3)
Hypertension, *n* (%)	54 (59)
Diabetes, *n* (%)	29 (32)
Coronary disease, *n* (%)	21 (23)
Stroke/TIA, *n* (%)	10 (11)
LVEF, %; median (IQR)	55 (45–60)
LAD, mm; median (IQR)	41 (38**‐**45)
CHADS‐VA score; median (IQR)	2 (1–3)
Anticoagulation with non‐vitamin K antagonist, *n* (%)	91 (100)
Antiplatelet therapy, *n* (%)	23 (25)

*Note:* Continuous variables are presented as mean (SD) or median (IQR) where appropriate, whereas categorical parameters are counts and percentages. Abbreviations: AF, atrial fibrillation; BMI, body mass index; EHRA, European Heart Rhythm Association; LAD, left atrial diameter; LVEF, left ventricular ejection fraction; TIA, transient ischemic attack.

The majority of the procedures (78%) were performed under general anesthesia. Procedure time and left atrial dwell time were 65 (52–80) min, and 27 (24–32) min, respectively. No major complications occurred. A detailed description of the procedural characteristics is shown in Table 2.

**Table 2:** Procedural characteristics.

Parameter	Total number (*n* = 91)
General anesthesia, *n* (%)	71 (78)
Procedure time, min; median (IQR)	65 (52–80)
Left atrial dwell time, min; median (IQR)	27 (24–32)
Typical LPV anatomy, *n* (%)	59 (65)
PF applications in LPV, *n*; median (IQR)	8 (8–8)
Fluoroscopy time, min; median (IQR)	12 (8–15)
Dose area product, uGym^2^; median (IQR)	455 (231–886)
Absorbed dose, mGy; median (IQR)	60 (15–101)
Major complications, *n* (%)	0 (0)
Minor complications, *n* (%)	2 (2)

*Note:* Continuous variables are presented as mean (SD) or median (IQR) where appropriate, whereas categorical parameters are counts and percentages. Abbreviations: LPV, left‐sided pulmonary vein; PF, pulsed‐field.

**Figure 2:**
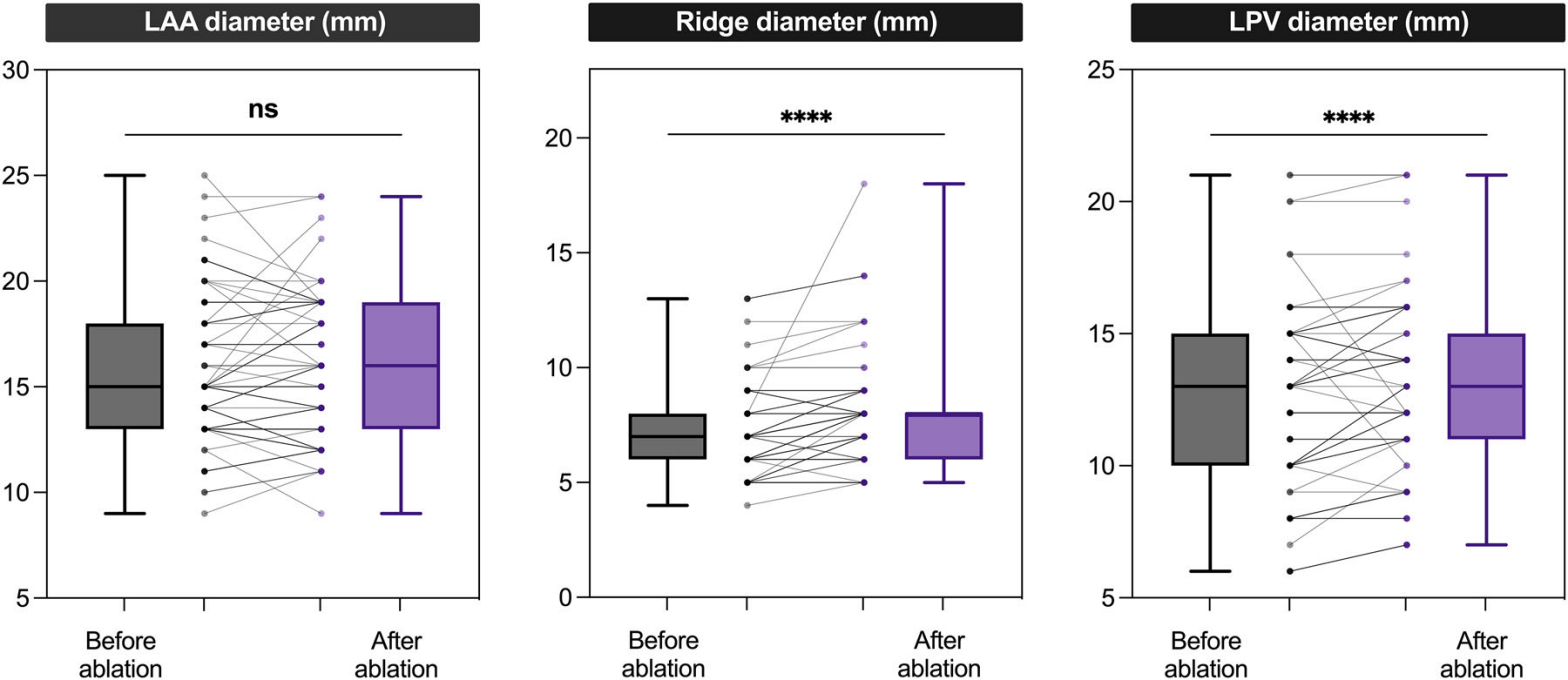
Echocardiography measurements of the left atrial appendage, ridge, and left pulmonary vein before and after the ablation in the whole study population. Darker shades indicate a higher number of patients. Parameters before and after the ablation were analyzed using the Wilcoxon signed‐rank test. LAA, left atrial appendage; LPV, left‐sided pulmonary vein; ns, not significant; **** = *p* < 0.0001.

Echocardiography measurements were performed with ICE in 41 and TOE in 50 cases. The diameter of the LAA was not different before and after the PVI (*p* = 0.756). On the other hand, the diameter of the left atrial ridge and the left‐sided pulmonary vein was larger after ablation compared to the diameter before PVI (*p* < 0.0001 for both); see Table 3 and Figure 2. Furthermore, we analyzed the LAA diameter for all three centers separately, which was nonsignificant in all three centers. We also compared if there is any difference between LAA measurement results when analyzing TOE and ICE modalities separately. Both for TOE and ICE modalities, all results (LPV, LAA, ridge before vs. after) remained the same as in the whole population.

**Table 3:** Echocardiography measurements before and after the ablation.

Parameter	Before ablation	After ablation	p‐value
Left atrial appendage, mm; median (IQR)	15 (13‐18)	16 (13‐19)	0.756
Left atrial ridge, mm; median (IQR)	7 (6‐8)	8 (6‐8)	< 0.0001
Pulmonary vein, mm; median (IQR)	13 (10‐15)	13 (11‐15)	< 0.0001

Continuous variables are presented as median (IQR), whereas categorical parameters are counts and percentages. Parameters before and after the ablation were analyzed using the Wilcoxon signed‐rank test.

Repeated measurements by a separate researcher demonstrated good interobserver variability for all three structures: ICC = 0.95 (95%CI = 0.91–0.98) for LAA, 0.94 (95%CI = 0.88–0.97) for the left atrial ridge, and 0.93 (95%CI = 0.86–0.97) for LPV.

## 4 Discussion

### 4.1 Main Finding

Pulsed‐field ablation‐based PVI with the pentaspline catheter results in a significant but clinically nonrelevant change in the pulmonary vein and left atrial ridge anteroposterior diameter. PVI with PFA does not affect the anteroposterior diameter of the LAA.

### 4.2 Edema Formation During Pulsed‐Field Ablation

Pulsed‐field ablation‐based PVI with the pentaspline catheter requires repeated applications to ensure the optimal lesion creation [17]. Moreover, the optimal workflow includes further ablations after rotating the device and repeating the same in two catheter configurations (e.g., basket and flower) [18]. The number of PF applications may contribute to a more pronounced edema formation.

The edema formation after PFA is present, but our knowledge of its extent is limited. Nakatani et al. investigated tissue characteristics with preablation, immediate postablation, and 3‐month cardiac magnetic resonance imaging, and they showed that the PFA‐induced edema is about 20% smaller compared to the edema caused by thermal ablations [19]. Another study analyzed peri‐esophageal edema after PFA and thermal ablation and again found a lower rate of edema formation with pulsed‐field energy [20]. Animal studies showed that edema formation after PFA seems similar to radiofrequency ablation, but they also noted that tissue swelling is more pronounced in animals than in humans [21].

Edema formation caused by PFA may progress within a few hours, as shown by Kawamura et al. They found that edema is more extensive at 4 h compared to 1 h after the PFA [22]. Although left‐sided PVs were ablated first in our study, the postablation measurements of the structures happened less than 30 min after the PFA. The changes observed in our population after ablation may become more pronounced if measured at later time points.

### 4.3 Concomitant PVI and LAAO

In certain AF patients, combined AF ablation and LAAO may be indicated to reduce both stroke risk and AF burden. As PVI and LAAO are invasive procedures, they carry a specific risk of vascular access site complications, pericardial effusion, or silent thromboembolic events related to the LA access. Previously, due to the length of PVI, the two procedures were performed separately. Nowadays, with the development of catheter ablation technologies, shorter procedural durations are achievable, making concomitant PVI and LAAO feasible [8].

The recently published OPTION trial was the first randomized controlled trial comparing the LAAO versus anticoagulation after PVI. This study concluded that LAAO was associated with a lower risk of non‐procedure‐related major or clinically relevant nonmajor bleeding than oral anticoagulation and was non‐inferior to oral anticoagulation for a composite of death from any cause, stroke, or systemic embolism at 36 months [9].

ICE is useful to detect LAA thrombus and guide AF ablation [23, 24]. Moreover, data regarding the safety and efficacy of ICE‐guided LAAO procedures are also robust [25, 26]. In our current study, where ICE was used, it guided many aspects of the PFA procedures, starting from the transseptal puncture to the visualization of optimal PV alignment, catheter‐tissue contact, and the LAA size measurements.

**Figure 3:**
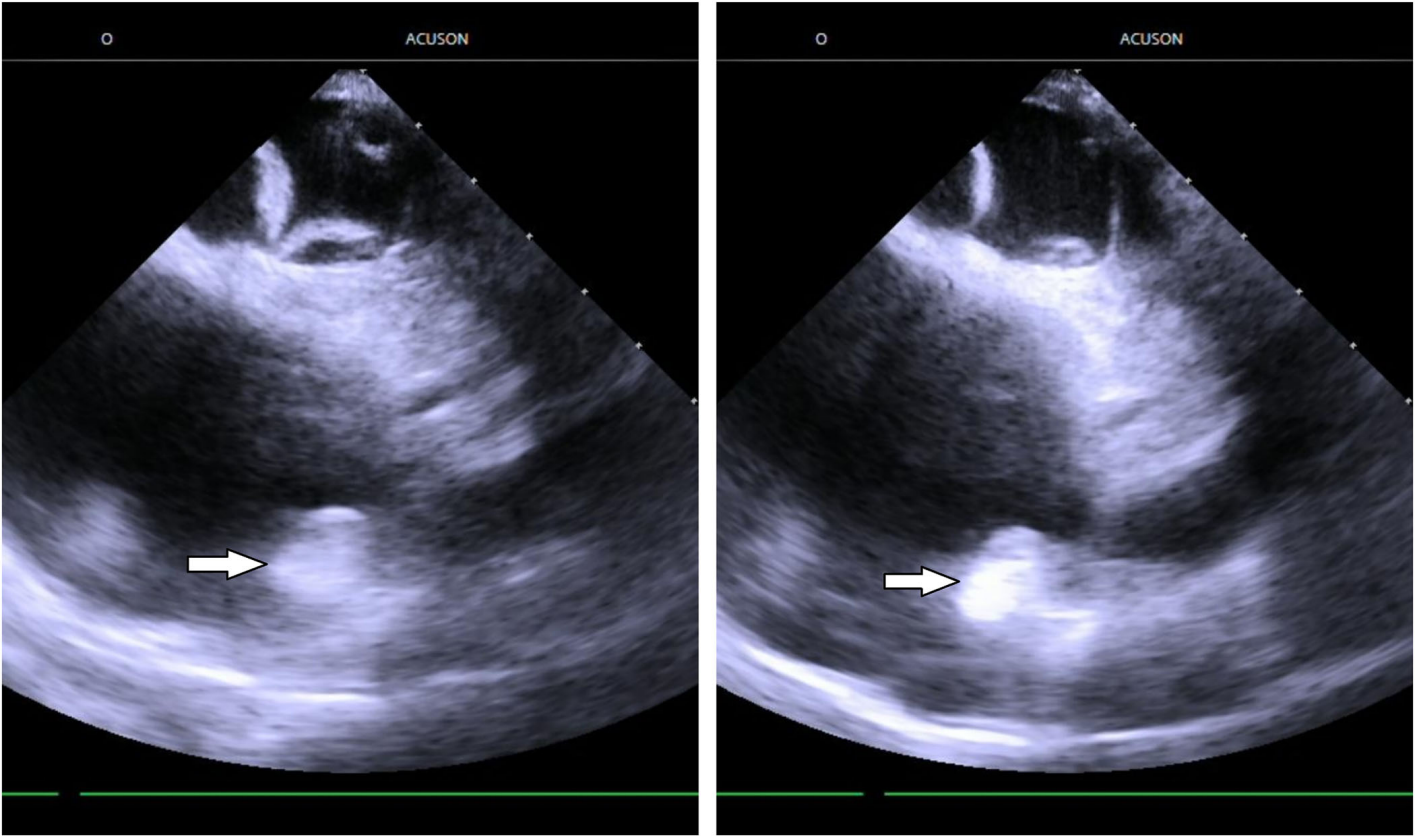
Increase in echogenicity of the left atrial ridge after ablation. Left panel: before ablation, right panel: after ablation. White arrows point to the ridge.

A recent case report by Gaggiotti et al. raised concerns about a potential risk of concomitant PFA‐based PVI and LAAO. They reported a PVI with a pentaspline PFA catheter, followed by LAAO using a WATCHMAN FLX device. After PVI, acute edema of the left atrial ridge was observed, yet a 27 mm LAAC device was successfully implanted. Follow‐up TOE after 6 weeks showed the resolution of the edema and tilting of the LAAO device without leakage. Importantly, at the 6‐month follow‐up, no stroke or bleeding events were recorded [14]. The ablation protocol was 10 PF applications per PV. On the contrary, our current results show that there is no clinically relevant change in LAA diameter using eight applications per PV. Notably, we observed an increased echogenicity (e.g., pronounced whitening, see Figure 3) of LA ridge tissue after PF applications with a mean increase in its dimension by 1 mm. It seems that despite statistical significance, this has no clinical relevance due to the lack of influence on the choice of size of the LAAO device. Notably, in one patient, we observed a 10 mm increase in the diameter of the ridge following 10 PF applications. This case highlights the importance of intraprocedural echocardiographic assessment to monitor edema formation at the ridge after ablation. We want to emphasize that our findings are limited to LAA diameter measurements and that extrapolation of these results to concomitant PVI plus LAAO feasibility requires dedicated future studies.

The PVI with the pentaspline catheter may have several advantages when considering a combined procedure with LAAO, e.g., shorter left atrial dwell times during the PVI, same transseptal sheath size for both devices. Obviously, the feasibility of concomitant PFA‐based PVI and LAAO must be confirmed by future dedicated clinical studies.

### 4.4 Limitations

We acknowledge that our study has certain limitations. Although it is a multicenter study, the overall number of included patients is moderate. Potentially, there is subjectivity in the echocardiography measurements, but repeated ICE measurements showed good interobserver agreement. The LAA size was only assessed by its anteroposterior diameter, but no volumetric measurements were performed. Notably, the potential change in LAA size may only occur in this measure by a change in the LAA ridge's thickness. The ICE measurements were performed shortly after the ablation of the left‐sided PVs, as the left atrial dwell time was around half an hour. Since edema formation after PFA may progress over time, these results cannot be translated to longer procedures (e.g., with additional left and right atrial ablations). Two different echocardiographic modalities (TOE and ICE) were used in this study. While stratified analyses demonstrated consistent findings within each modality, the lack of cross‐validation means that results from TOE and ICE cannot be directly compared. Finally, as no LAAO was performed in the current patient population, no direct conclusion on the feasibility of concomitant PFA and LAAO can be drawn from our results, but further studies are needed to address this topic.

## 5 Conclusion

Left atrial appendage anteroposterior diameter does not change after pulsed‐field ablation‐based pulmonary vein isolation. The results were consistent in all three participating centers. Although there is a statistically significant change in the anteroposterior diameters of the left‐sided pulmonary vein and the left atrial ridge, it does not seem clinically relevant. Intraprocedural echocardiography should be used to monitor tissue swelling, as significant edema formation on the LAA ridge might occur. Our findings may be useful in laying the groundwork for future prospective studies investigating combined PFA‐based PVI and LAAO procedures.

## Conflicts of Interest

The authors declare no conflicts of interest.

## Supporting information

Supplementary material.

## Data Availability

The data that support the findings of this study are available on request from the corresponding author. The data are not publicly available due to privacy or ethical restrictions. The data underlying this article cannot be shared publicly due to privacy/ethical reasons (General Data Protection Regulation). The data will be shared on reasonable request to the corresponding author.
