# Supplementary material for: Impact of Pulsed‐Field Ablation on the Left Atrial Appendage Diameter—Insights From Intra‐Procedural Echocardiography
Source: J Cardiovasc Electrophysiol. 2025 Sep 19;36(12):3149–55. doi: 10.1111/jce.70111 (PMC12697231; doi:10.1111/jce.70111)
Supplement: Supplementary file 1 — Supplementary material. [file JCE-36-3149-s001.docx]

**Supplementary material**

Supplementary table 1. Inclusion and exclusion criteria

| **Inclusion Criteria** | **Exclusion Criteria** |
| --- | --- |
| - Symptomatic paroxysmal, persistent, or long-standing persistent AF | - Contraindications to ablation |
| - Voluntary consent to participate | - Prior cardiac surgery involving the left atrium (e.g., mitral valve repair, LAA ligation) |
| - Age >18 years | - Pregnancy, active malignancy, life expectancy <1 year |
|  | - Prior left atrial ablation |
|  | - Left atrial thrombus detected before ablation (confirmed by TOE or ICE) |
|  | - Significant valvular heart disease (moderate/severe mitral or aortic stenosis/regurgitation) |
|  | - Reversible cause of AF |
